# Association between public media and trends in new acute coronary syndrome presentations during the first COVID‑19 wave in the Netherlands

**DOI:** 10.1007/s12471-021-01603-5

**Published:** 2021-07-29

**Authors:** L. Derks, H. N. Sturkenboom, M. Zaal, S. Houterman, P. Woudstra, R. A. Tio, H. P. A. van Veghel

**Affiliations:** 1 Netherlands Heart Registration, Utrecht, The Netherlands; 2 Department of Cardiology, Catharina Hospital, Eindhoven, The Netherlands; 3 Dutch Hospital Data, Utrecht, The Netherlands; 4 Department of Cardiology, MCL, Leeuwarden, The Netherlands

**Keywords:** Acute coronary syndrome, COVID‑19, Non-ST-segment elevation myocardial infarction, ST-segment elevation myocardial infarction, Unstable angina

## Abstract

**Background:**

We aimed to evaluate the association between public media and trends in new presentations of acute coronary syndrome (ACS) during the first wave of the coronavirus disease 2019 (COVID‑19) in the Netherlands.

**Methods:**

New ACS presentations per week in 73 hospitals during the first half of 2019 and 2020 were retrieved from the national organisation Dutch Hospital Data and incidence rates were calculated. Stratified analyses were performed by region, type of ACS and patient characteristics.

**Results:**

After the first confirmed COVID‑19 case and during lockdown, numbers declined by up to 41% (95% confidence interval (CI): 36–47%) compared to 2019. This reduction was more pronounced for non-ST-segment elevation myocardial infarction (NSTEMI) (48%; 95% CI: 39–55%) and unstable angina (UA; 50%; 95% CI: 40–59%) than for STEMI (34%; 95% CI: 23–43%). There was no association between ACS and COVID‑19 incidence rate per region. After the steep decline, a public campaign encouraged patients not to postpone hospital visits. Numbers then increased, without a rebound effect. Trends were similar irrespective of sex, age or socio-economic status. During the outbreak, compared to coronary artery bypass graft procedures, relatively more (acute) percutaneous coronary interventions for NSTEMI and UA were performed.

**Conclusion:**

New ACS presentations decreased by up to 41%. Lockdown measures and public campaigns, rather than COVID‑19 incidence, were associated with significant changes in new ACS presentations. Even though causality cannot be established, this emphasises the role of the public media and healthcare organisations in informing patients to prevent underdiagnoses of ACS and associated health damage.

**Supplementary Information:**

The online version of this article (10.1007/s12471-021-01603-5) contains supplementary material, which is available to authorized users.

## What’s new?

- This is the first study to reliably investigate trends in new acute coronary syndrome (ACS) presentations during the COVID-19 pandemic in the Netherlands using data from a nationwide organisation that administers all Dutch in-hospital healthcare activities and related diagnoses.
- A significant reduction in new ACS presentations of up to 41% was observed during the first wave of COVID-19.
- Although the incidence of COVID-19 cases did not decrease, the number of new ACS presentations recovered after public campaigns.

## Introduction

The coronavirus disease 2019 (COVID-19) pandemic is a major burden affecting healthcare worldwide. After the first official case of COVID-19 in the Netherlands was reported on 27 February 2020 [1], numbers rose exponentially [2]. Compulsory measures and social distancing interventions in order to prevent further transmission were taken by the Dutch government and announced in the public media. Downregulation of regular healthcare to acute care only was inevitable due to the increase in hospitalisations of COVID-19 patients.

In several countries, a decline in presentations of acute coronary syndrome (ACS) was observed [3–14]. In cases of ST-segment elevation myocardial infarction (STEMI), timely revascularisation is warranted in order to prevent heart failure, mechanical complications, cardiogenic shock and even early death, and treatment delay is an independent negative predictor of survival [15–17]. Consequently, identifying and understanding the trends, scale, nature of and explanations for changes in new ACS presentations is of utmost importance, in order to prevent avoidable cardiovascular complications during comparable crisis situations.

The aim of the present study was to evaluate trends in new presentations and in-hospital treatment for ACS during the first wave of the COVID-19 pandemic in the Netherlands, and to investigate the association thereof with COVID-19 incidence rate, national governmental countermeasures and recommendations by the Netherlands Society of Cardiology (NVVC) announced in the public media.

### Methods

In-hospital healthcare activities, related diagnoses and patient characteristics are registered by physicians for 73 out of 74 Dutch hospitals in the Dutch National Hospital Care Registration (LBZ) [18].

All patients newly presenting with ACS, from 1 January until 30 June of both 2019 and 2020, were included for analyses on a weekly basis. Presentations were selected based on the following criteria: (1) registered by a cardiologist; (2) diagnosis of STEMI, non-STEMI (NSTEMI) or unstable angina (UA); (3) it concerns an initial presentation; and (4) the patient had no similar diagnosis in the 2 years preceding this study’s period of interest (2019 and 2020). Patient and hospital privacy was assured by aggregating data into seven regions, containing at least three hospitals, by a delegate of Dutch Hospital Data, the organisation that administers LBZ data [18, 19].

Data were divided into groups by (1) diagnoses (STEMI, NSTEMI, UA), (2) age (< 60, 60–79, ≥ 80 years), (3) sex, (4) socio-economic status (SES), constructed using postal codes following the methods described by Statistics Netherlands (CBS) [20], and (5) geographical region. Percutaneous coronary intervention (PCI) and coronary artery bypass graft (CABG) as in-hospital treatment for ACS were categorised as acute or non-acute, based on payment title codes assigned at the discretion of the physician (Electronic Supplementary Material, Table S1).

The number of confirmed COVID-19 cases per region was extracted from the data of the National Institute for Public Health and the Environment [2, 21]. Together with the 1 January 2020 provisional population numbers for each geographical region, which were retrieved from the CBS, the incidence rate of COVID-19-positive cases per week, per region, per 100,000 inhabitants was determined [21, 22]. Information and timing of stepwise lockdown measures introduced by the Dutch government were obtained online (Electronic Supplementary Material, Table S2) [23].

#### Statistical analysis

The number of ACS presentations and procedures (acute and non-acute PCI and CABG) performed per week were presented as counts. The percentage change per week was calculated using the equivalent week of 2019 as a reference. Differences between 2019 and 2020 were analysed using a Poisson distribution, to calculate 95% confidence intervals (95% CI). To determine statistically significant changes per week for distinct group of ACS presentations, percentages were compared using omnibus chi-square tests. *P*-values < 0.05 were considered statistically significant.

## Results

**Fig. 1:**
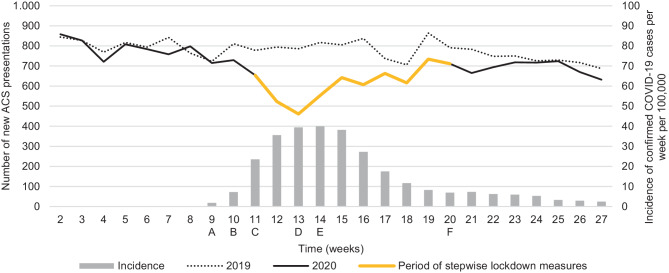
Total number of new presentations of acute coronary syndrome (*ACS*) per week in 2019 and 2020 together with incidence rate of confirmed COVID-19 cases per week per 100,000 population. *A* First COVID-19 case in the Netherlands: 27-02-2020. *B* Countermeasures in the south of the Netherlands: 06-03-2020. *C* Hygiene measures: 09-03-2020. Stay at home in case of symptoms, work from home, limit visits to vulnerable people: 12-03-2020. Declaration of COVID-19 pandemic: 11-03-2020. Broadcast about alarming situation in Italy: 12-03-2020. Social distancing (1.5 m), schools, bars/restaurants closed:15-03-2020. *D* Intelligent lockdown: stay at home, all gatherings prohibited: 23-03-2020. Public call: seek medical attention if experiencing potentially serious complaints: 27-03-2020. *E* Public call by the NVVC (Netherlands Society of Cardiology): call emergency services in case of cardiac symptoms: 01-04-2020. *F* Alleviation of countermeasures: 11-05-2020

**Fig. 2:**
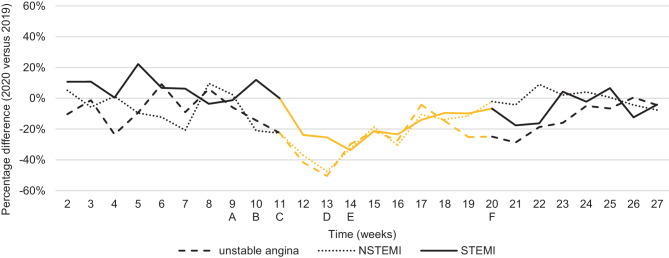
Percentage difference in new acute coronary syndrome presentations by diagnoses per week. *STEMI* ST-segment elevation myocardial infarction, *NSTEMI* non-STEMI. *A* First COVID-19 case in the Netherlands: 27-02-2020. *B* Countermeasures in the south of the Netherlands: 06-03-2020. *C* Hygiene measures: 09-03-2020. Stay at home in case of symptoms, work from home, limit visits to vulnerable people: 12-03-2020. Declaration of COVID-19 pandemic: 11-03-2020. Broadcast about alarming situation in Italy: 12-03-2020. Social distancing (1.5 m), schools, bars/restaurants closed: 15-03-2020. *D* Intelligent lockdown: stay at home, all gatherings prohibited: 23-03-2020. Public call: seek medical attention if experiencing potentially serious complaints: 27-3-2020. *E* Public call NVVC (Netherlands Society of Cardiology): call emergency services in case of cardiac symptoms: 01-04-2020. *F* Alleviation of countermeasures: 11-05-2020

Complete data concerning new ACS presentations and the procedures performed were available for 73 hospitals. The number of ACS presentations and the incidence rate of COVID-19 cases per week are presented in Fig. 1. Fig. 2 presents numbers of STEMI, NSTEMI and UA presentations, together with public announcements of countermeasures and healthcare campaigns during the COVID-19 outbreak. Before the onset of the COVID-19 outbreak in week 9 of 2020, no significant differences in new presentations were observed as compared to 2019. After the report of the first confirmed COVID-19 case in week 9, a significant decline was observed with a maximal decrease in presentations of 41% (95% CI: 36–47%) by week 13. The maximal reductions for NSTEMI (48%; 95% CI: 39–55%) and UA (50%; 95% CI: 40–59%) were comparable. The maximal decline in new STEMI presentations was 34% (95% CI: 23–43%) in week 14.

In parallel to the decline in presentations an increase in confirmed COVID-19 cases was observed. On 23 March (week 13) a nationwide lockdown was initiated. On 27 March (week 13), healthcare providers made a public call to all patients with serious complaints not to postpone hospital visits, followed by a second call to all patients with cardiac complaints made by the NVVC, on 1 April (week 14). Thereafter a gradual increase in presentations for STEMI and NSTEMI was observed, to levels comparable to the pre-COVID-19 period by week 23. For UA, a significant reduction was observed up to week 23 (16%; 95% CI: 3–27%). No significant rebound in new ACS presentations was observed. The first decrease in newly confirmed COVID-19 cases was registered in week 16. From week 20 onwards, alleviation of countermeasures was initiated. After the initiation or alleviation of different lockdown measures no significant changes in new ACS presentations were observed when compared to the previous week. Therefore, no association between the initiation or alleviation of lockdown measures and new ACS presentations could be established.

During the COVID-19 outbreak, no significant differences were observed in the reduction in new ACS presentations among the sexes (*p*-values = 0.41–0.83), SES (*p*-values = 0.06–0.99) or different age groups (*p*-values = 0.14–0.96). The decrease was more pronounced in region G (58%), in week 14, and lowest in region A (35%), in week 13. Although the incidence rate of COVID-19 was higher in region G, a similar reduction was observed in region C, where the incidence rate was relatively low (Tab. 1). Omnibus chi-square tests showed no significant differences among regions in the decline in the number of new ACS presentations per week during weeks 9 through 14 (*p*-values = 0.07–0.87).

**Table 1:** Percentage difference (2019 vs 2020) in new presentations of acute coronary syndrome per week, stratified by region and patient characteristics

Week			9	10	11	12	13	14
Geographical location^a^ (mean incidence of confirmed COVID-19 cases per week per 100,000)	A	(19.9)	2%	−19%^b^	−28%^b^	−10%	−35%^b^	−21%^b^
	B	(23.4)	−11%	−34%^b^	−7%	−34%^b^	−40%^b^	−28%^b^
	C	(21.7)	15%	−18%	26%	−44%^b^	−53%^b^	−41%^b^
	D	(25.7)	−3%	2%	−23%^b^	−31%^b^	−41%^b^	−22%^b^
	E	(21.0)	−11%	−6%	−21%^b^	−46%^b^	−39%^b^	−38%^b^
	F	(32.0)	25%	3%	−7%	−44%^b^	−48%^b^	−26%^b^
	G	(36.5)	−22%	3%	−13%	−23%	−35%^b^	−58%^b^
Sex	Men		0%	−7%	−14%^b^	−35%^b^	−40%^b^	−32%^b^
	Women		−4%	−15%^b^	−20%^b^	−32%^b^	−45%^b^	−33%^b^
Age category	< 60 years		17%	−10%	−7%	−30%^b^	−40%^b^	−38%^b^
	60–79 years		−7%	−9%	−18%^b^	−32%^b^	−40%^b^	−32%^b^
	≥ 80 years		−8%	−13%	−22%^b^	−46%^b^	−49%^b^	−20%^b^
Socio-economic status	Lowest		19%	−5%	−18%	−37%	−46%	−33%
	Below average		−15%	−16%	−20%	−32%	−29%	−30%
	Average		15%	−3%	−16%	−30%	−40%	−30%
	Above average		−9%	−7%	−12%	−33%	−45%	−35%
	Highest		−19%	−22%	−11%	−38%	−46%	−31%

^a^Geographical location: *A* Groningen, Friesland, Drenthe and Overijssel, *B* Gelderland, *C* Utrecht and Flevoland, *D* Noord Holland, *E* Zuid Holland and Zeeland, *F* Noord Brabant and *G* Limburg

^b^Statistically significant reduction compared to equivalent week in 2019

In parallel with the reduction in STEMI presentations (34%) in week 14, a similar decrease in acute PCI (37%; 95% CI: 26–47%), non-acute PCI (38%; 95% CI: 14–57%) and CABG (44%; 95% CI: 30–82%) procedures for STEMI was observed. In week 13, the reduction in NSTEMI presentations (48%) was not accompanied by a similar decrease in acute PCI procedures (29%; 95% CI: 13–43%), while the reductions in non-acute PCI (62%; 95% CI: 46–74%) and CABG (62%; 95% CI: 37–78%) procedures were more in line with the expected. No significant decrease in acute PCI procedures for UA was observed, even though UA presentations declined by up to 50% in week 13. There were, however, significant reductions in non-acute PCI and CABG procedures for UA. The decrease in CABG procedures (74%; 95% CI: 50–88%) were more than expected in relation to the maximum reduction in UA presentations. In conclusion, proportionally more acute PCI procedures for NSTEMI and more PCI (acute and non-acute) procedures for UA were observed during the outbreak as compared to 2019 (Tab. 2).

**Table 2:** Percentage difference (2019 vs 2020) in in-hospital treatment for new presentations of acute coronary syndrome per week

Week		9	10	11	12	13	14
STEMI	Acute PCI	−6%^a^	16%	0%	−14%^a^	−21%^a^	−37%^a^
	Non-acute PCI	−29%	31%	11%	−8%^a^	−48%^a^	−38%^a^
	CABG	−19%	−50%^a^	−21%	−57%^a^	−39%^a^	−44%
NSTEMI	Acute PCI	−2%	−14%	−36%^a^	−31%^a^	−29%^a^	9%
	Non-acute PCI	−30%	−16%	−16%	−15%	−62%^a^	−48%^a^
	CABG	3%	−23%	2%	−45%^a^	−62%^a^	−64%^a^
UA	Acute PCI	−13%	−11%	−18%	−21%	−17%	35%
	Non-acute PCI	2%	51%	−12%	−48%^a^	−36%^a^	−44%^a^
	CABG	−11%	−26%	7%	−12%	−74%^a^	−81%^a^

*CABG* coronary artery bypass graft, *NSTEMI* non-ST-segment elevation myocardial infarction,* PCI* percutaneous coronary intervention, *STEMI* ST-segment elevation myocardial infarction, *UA* unstable angina

^a^Statistically significant reduction compared to equivalent week in 2019

## Discussion

In the present study, a significant reduction in new ACS presentations of up to 41% was observed during the COVID-19 pandemic as compared to the equivalent period in 2019. The decrease was more pronounced for NSTEMI and UA than for STEMI, in accordance with observations in Italy (54% vs 26%) England (49% vs 29%) and Austria (53% vs 31%) [4, 7, 24]. Despite the high incidence of COVID-19, an increase in ACS presentations was observed from week 14 onwards.

The first significant decrease in new ACS presentations was observed in the week after the first confirmed case of COVID-19 and before national countermeasures were announced. Communication about the first COVID-19 case and reports of rapidly increasing numbers of COVID-19-positive cases clearly preceded the change in medical help-seeking behaviour.

There are several potential explanations for the observed decrease in new ACS presentations. Fear of contracting COVID-19 might have caused patients to postpone hospital visits as described previously, whilst decreased physical activities or diminished stress during the lockdown could have contributed to the occurrence of fewer ACS cases [3, 25, 26]. The simultaneous reduction in air pollution from week 9 onwards reflects a sudden decrease in road transport (and economical) activities [27]. Furthermore, the reduction in air pollution in itself might have contributed to the decrease in ACS, since it is a known trigger for myocardial infarction [13]. Lastly, patients who normally developed ACS potentially developed severe COVID-19 disease; in such cases, cardiac complaints might have gone undetected or people may have died from the disease before developing ACS. However, these potential contributing factors do not fully explain the observed trend in presentations during the COVID-19 pandemic in the Netherlands.

In our study an increase in ACS presentations was observed following public calls by healthcare professionals in week 13. This effect was also described after a public call by the British Heart Foundation and a British Cardiovascular Society publicity campaign [3]. In the United States and New Zealand the aforementioned association was less explicitly described [28–30]. The reduction in presentations and the increase after the public calls to patients might be explained by the effect of the public media on patients’ behaviour. According to Wallack et al. (public) media can be used to achieve ‘advocacy’, to create a shift in public opinion, which can be useful in health-promotion campaigns [31]. Renaud et al. provided a conceptual framework of how the media can shape socially constructed understandings of health [32]. In this framework of health-promotion interventions, in order to achieve advocacy, initiation of changes in health-related norms determined by the public (‘receptors’) must be promoted by agents (i.e. healthcare specialists) involved in the changing process. During the COVID-19 outbreak, public health messages promoted by healthcare specialists and governmental delegates, together with updates on epidemiological facts, death rates, and national countermeasures aimed at containing the virus, might have caused a shift in public ‘norms’ towards general fearfulness, resulting in patients avoiding social interactions and postponing hospital visits. The subsequent public calls by healthcare specialists may have helped to overcome the fear of infection. Thus, medical professionals play a public role in achieving ‘advocacy’ and are thus pivotal in preventing unwanted help-seeking delays during unprecedented events, such as the COVID-19 pandemic.

The likelihood of an effect of public media on patients’ behaviour, and thus the observed trends, is further supported by similar changes in ACS presentations per patient group and among regions, even though incidence rates of COVID-19 varied per region. This observation was confirmed in Italy and France, which had no regional differences in ACS presentations, while large differences in incidence rates were seen between the northern and southern parts of the respective countries [7, 12].

Accompanied by the decrease in new ACS presentations, we also observed a relative increase in acute PCIs for NSTEMI and PCIs for UA, which was most likely the result of downregulation of regular healthcare, with postponement of elective procedures, resulting in better availability catheterisations staff and facilities. Furthermore, the large numbers of COVID-19 patients caused capacity problems for operation facilities and intensive care units for postoperative care, making CABG a less favourable treatment option. More research regarding treatment prevalence and long-term outcomes of ACS during the COVID-19 pandemic is therefore warranted.

Finally, no rebound in the number of new ACS presentations was observed after the initial decline and/or after termination of the lockdown measures. Potentially, the reduction in new ACS presentations reflects underdiagnoses of true cases. This was further substantiated by an observed increase in out-of-hospital cardiac arrest during the COVID-19 pandemic [33, 34]. More awareness concerning possible missed ACS cases and consequently new-onset heart failure and mortality during and after the pandemic is warranted. We therefore suggest prospective research concerning new-onset heart failure. Also, in order to identify the need for an intervention at an early stage, we suggest that national data on ACS admissions should be tracked in a timely fashion and made available to public health agencies. We emphasise the impact of public media and the pivotal role played by medical professionals in preventing unwanted delays in help-seeking during the ongoing pandemic or comparable unprecedented events.

When interpreting our findings, some limitations should be noted. First, for this study, data of the LBZ, provided by healthcare providers rather than a clinical register, were used. However, previous research showed a high level of comparison between LBZ data and clinical registration of ACS patients [35]. Furthermore, with almost complete national coverage, and by comparing 2020 data with 2019 data, we consider that the presented numbers are reliable. Secondly, only a few stratifications could be made to identify potentially vulnerable groups. Finally, it was not possible to investigate the potential impact on outcomes by using the current data or to deduct causality between the observed and described associations.

In conclusion, a significant reduction in new ACS presentations was observed during the COVID-19 pandemic. The reduction was more pronounced for UA and NSTEMI than for STEMI. Under-diagnosis of ACS, the postponement of hospital visits and the effect of the public media on patients’ behaviour are the most likely explanatory factors for the observed changes in new ACS presentations. Although causality cannot be established, additional awareness regarding the possible effect of communication by (societies of) healthcare providers and public media on new ACS presentations during the ongoing pandemic or comparable unprecedented events is warranted.

## Supplementary Information

Supplementary Material Table S1: Categorization of payment title codes for acute and non-acute in-hospital treatment options for Acute Coronary Syndrome. Supplementary Material Table S2: Timeline of stepwise lockdown measures.
