# Supplementary material for: Association between public media and trends in new acute coronary syndrome presentations during the first COVID‑19 wave in the Netherlands
Source: Neth Heart J. 2021 Jul 29;29(11):577–83. doi: 10.1007/s12471-021-01603-5 (PMC8320720; doi:10.1007/s12471-021-01603-5)
Supplement: Supplementary file 1 — Supplementary Material Table S1: Categorization of payment title codes for acute and non-acute in-hospital treatment options for Acute Coronary Syndrome. Supplementary Material Table S2: Timeline of stepwise lockdown measures. [file 12471_2021_1603_MOESM1_ESM.docx]

**Supplementary files**

**Supplementary Table 1** Payment titles per category

| **Procedure** | **Payment title code** | **Payment title description** |
| --- | --- | --- |
| Coronary artery bypass graft | 033104 | Aortocoronary bypass surgery with only venous graft(s) and/or plastic material |
|  | 033105 | Aortocoronary bypass surgery with 1 arterial graft, including any venous graft(s) and/or plastic material |
|  | 033106 | Aortocoronary bypass surgery with 2 arterial grafts, including any venous graft(s) and/or plastic material |
|  | 033107 | Aortocoronary bypass surgery with 3 or more arterial grafts, including any venous graft(s) and/or plastic material |
|  | 033098 | Ascending aorta replacement without circulatory arrest |
| Acute percutaneous coronary intervention | 033238 | Acute percutaneous transluminal coronary angioplasty (PTCA) to correct/remove coronary stenoses |
| Non-acute percutaneous coronary intervention | 033231 | PTCA of one branch to correct/remove coronary artery stenoses |
|  | 033232 | PTCA of multiple branches or main stem to correct/remove coronary artery stenoses |
|  | 033233 | PTCA for lifting/removing chronic occlusion of coronary arteries |
|  | 033234 | PTCA with passage coronary artery graft |

**Supplementary Table 2** Information and timing of stepwise lockdown measures

| **Week** | **Date** | **Event** |
| --- | --- | --- |
| 9 | 27 February 2020 | First confirmed COVID-19 case in The Netherlands |
| 10 | 6 March 2020 | Start of countermeasures in southern region Netherlands: stay home when experiencing symptoms |
| 11 | 9 March 2020 | Start of nationwide hygiene measures |
|  | 11 March 2020 | WHO declares COVID-19 as a pandemic |
|  | 12 March 2020 | Start nationwide countermeasures: stay home when experiencing symptoms, work from home if possible, avoid contact with vulnerable people |
|  | 12 March 2020 | Broadcast by Italian nurse who raises alarm about COVID-19 situation in Italy |
|  | 15 March 2020 | Social distancing (1.5 m), closure of schools, bars, and restaurants |
| 12 | 16 March 2020 | Speech by Prime Minister Rutte |
|  | 17 March 2020 | Global travel restrictions |
|  | 20 March 2020 | Speech by King Willem Alexander |
| 13 | 23 March 2020 | Start of intelligent lockdown: stay at home if possible, all gatherings prohibited, ban on assembly, gyms and hairdressers closed, public transport only for essential personnel. |
|  | 27 March 2020 | Public call by NOS (Dutch news) to seek medical attention when experiencing potentially serious complaints |
| 14 | 1 April 2020 | Public call by NVVC (The Dutch association of Cardiology) to call the emergency services immediately in the case of experiencing cardiac symptoms |
| 20 | 11 May 2020 | Start of alleviation of countermeasures: avoid busy places, instead of staying at home; partial re-opening of schools |
